# Healthcare professionals' perceptions of a digital parental support, *Childbirth Journey*, constructed as a serious game—An intervention study

**DOI:** 10.3389/fdgth.2023.1141350

**Published:** 2023-04-04

**Authors:** Caroline Bäckström, Rajna Knez, Margaretha Larsson

**Affiliations:** ^1^ Department of Caring Science, University of Borås, Borås, Sweden; ^2^ School of Health Sciences, University of Skövde, Skövde, Sweden; ^3^ Research Group Family Centered Health (FamCeH), University of Skövde, Skövde, Sweden; ^4^ Skaraborgs Hospital, Lövängsvägen, Skövde, Sweden

**Keywords:** digital health, application, parents, pregnancy, midwife, digitalisation, antenatal education, qualitative methods

## Abstract

**Background:**

Globally, the digital sources developed and available in antenatal care differ, and infrastructure challenges may impede the further development of such sources. Challenges accompanying digital developments can include the commonly occurring high workload, which affects healthcare professionals' ability to acquire professional knowledge about how to best support parents in using digital sources. Including healthcare professionals in the development process of digital sources may increase the likelihood that such sources will be adopted and employed by these professionals in their future care work. Therefore, the present research explored healthcare professionals' perceptions of the digital support intervention *Childbirth Journey,* which was constructed as a serious game for expectant parents.

**Methods:**

Data were collected through semi-structured focus-group interviews with 11 midwives at antenatal, labour and postnatal clinics as well as with child healthcare nurses. Prior to the interviews, all participants were provided the intervention, *Childbirth Journey,* which is a serious game in a mobile application format consisting of two distinct parts: (1) a story-driven game and (2) a *Knowledge Portal*. The data were analysed using phenomenographic methods.

**Results:**

The perceptions of *Childbirth Journey* by healthcare professionals, midwives and child healthcare nurses are presented in four descriptive categories: extended professional support, trustworthy contents, diversity or individuality, and both appealing and in need of development.

**Conclusions:**

Current study revealed that *Childbirth Journey* may be utilised as a digital support for parents, allowing healthcare professionals to offer a digital solution as a complementary support to standard, face-to-face meetings with caregivers. However, the research results also revealed that some elements of *Childbirth Journey* must be improved, thereby representing a main contribution of this study: insights into how to better develop digital tools under the umbrella of health care. Thus, we conclude that in order to create sustainable and safe digital care solutions that function as trustworthy professional supports instead of technical products that risk harming users, the perspectives of both patients and healthcare professionals should be considered in the exploration and development of these solutions.

## Background

Digital health interventions are categorised by the World Health Organization (WHO) as “the different ways in which digital and mobile technologies are being used to support health systems” (1). Such interventions have been described as having the potential to improve the efficiency and effectiveness of the health system and its functions, such as distributing health information, among others (2). However, digital health interventions are not always effective in health care—sometimes, healthcare clients have poor access to cellular networks, the Internet or mobile devices, among other issues (3).

A Canadian study of how new parents experience digital technology highlighted the degree to which parents need these technologies to assist them in various aspects of their daily life, such as in making decisions concerning their parenting. Conversely, the parents expressed concerns about their personal patterns of using the technology because they felt it might disseminate unreliable information (4). The fact that many parents perceive the Internet as consisting of unreliable information has been stressed in several previous studies (4, 5).

A systematic review revealed that digital sources give expectant parents improved access to information and more opportunities to extend their social connections. Consequently, digital sources can bolster expectant parents' capacity to understand and adapt to parenthood and improve their health and well-being during the parental transition. That said, the authors of this review stressed the importance of conducting multi-sectorial collaborations and coordination between different organisations, such as antenatal, labour and postnatal care, to ensure the continuous dissemination of trustworthy information for parents (6).

In Sweden, as well as in several other countries, parental supports are included in midwifery work within antenatal care. Examples of such parental supports are preparations for labour and birth, as well as for parenting and breastfeeding (7). Within the WHO's definition of antenatal care (8), it is specified that such care should be provided to pregnant women by skilled healthcare professionals to ensure the best health conditions for both the mother and foetus. Antenatal care includes, among other services, the prevention and management of pregnancy-related diseases as well as health education and health promotion. In Sweden, the National Board of Health and Welfare (*Socialstyrelsen*) has claimed that antenatal care should be sensitive to the needs of parents and that alternative forms of support should be offered, such as digital educational materials (7).

Globally, the digital sources developed and available in midwifery care differ, and infrastructure challenges may impede their further development (9). Other challenges to the development and implementation of digital sources in midwifery care include the commonly occurring high workload, which affects the ability of midwives to acquire professional knowledge about how to best support women and their families in the use of digital sources. Hence, it has been asserted that organisational processes should be redesigned to meet the digital technology needs of women and their families, as well as those of midwives (10). It is therefore critical to invest in digital midwifery and digital development as doing so would benefit a wide range of populations, from women and their families to midwives and midwifery care organisations—and, of course, our communities at large (11).

Recent meta-review conclude that serious game is rapidly growing area that can be used to promote health behaviour (12). A digital parental support tool, *Childbirth Journey*, was developed as a serious game in Sweden through the collaborative efforts of researchers, clinical midwives, heads of midwifery care and a game company. *Childbirth Journey* was developed in a mobile application format and made available as an intervention for expectant and new parents. *Childbirth Journey* includes medical information relevant for parental support, which is provided through a “*Knowledge Portal”* containing information and references to external websites of interest to expectant parents. Furthermore, *Childbirth Journey* consists of a story-driven, 3D game in which avatars representing the birthing woman, her partner and healthcare professionals are illustrated in various birth scenarios. The player (i.e., the expectant parent) can influence the scenarios such that the story events unfold differently depending on which options are chosen. Extant research has shown that parents perceive the information provided *via Childbirth Journey* as easily accessible and reliable. When played together—i.e., by both members of the parental couple—*Childbirth Journey* could facilitate mutual understanding and stimulate productive discussions. However, several changes to the existing version of *Childbirth Journey* were proposed by parents, mostly due to the game's design and animations (13).

As Stevenson et al. (14) concluded in their research on developing a digital communication application for maternity care, involving midwives in the development of digital sources may guide the design and development of content in ways that better address specific user needs in practice. Besides, including midwives in the development of digital communication applications and related tools and interventions increases the likelihood that they will adopt and employ them in their future work within maternity care. Therefore, it was considered valuable to conduct research on midwives' perceptions of *Childbirth Journey* as a digital parental support tool. Similarly, it was considered valuable to conduct research on other healthcare professionals who work with new parents—specifically, their perceptions of *Childbirth Journey*—for the purpose of broadening knowledge regarding how various professions perceive the supportive dimensions of digital solutions for expectant and new parents. Hence, the aim of this study was to explore healthcare professionals' perceptions of *Childbirth Journey* as a digital tool, constructed as a serious game, that disseminates medical information for parental support.

## Methods

The present study was part of a larger research project, *Digital parental support* [please see the study protocol by Bäckström et al. (15)]*,* which involves mixed methods representing both inductive and deductive approaches. This study was retrospectively registered [(02/10/2020)] within the ISRCTN with ID: [(ISRCTN18017741)]. Within the larger research project [i.e., study protocol (15)], the current study is referred to as “*study C”* and includes an intervention, *Childbirth Journey,* which is offered to healthcare professionals at antenatal clinics, as described further below. The present study aimed to answer the following research question: How do healthcare professionals perceive *Childbirth Journey* as a digital support tool for parents with regard to pregnancy, childbirth and parenthood? In the present paper, the title “healthcare professionals” is used as an umbrella term for both midwives and child healthcare nurses.

The data were collected through interviews with healthcare professionals who chose to participate in the study. This study included an explorative design, a qualitative method, an inductive approach and a phenomenographic method for data analysis. Originally, phenomenography was developed within the pedagogical tradition, but it is now commonly used in nursing research as well. Via phenomenography, the various ways in which phenomena are understood can be distinguished (16).

### Intervention

Midwives at antenatal, labour and postnatal clinics as well as child healthcare nurses were invited to participate in the study. All healthcare professionals who agreed to participate in the study were provided with the *Childbirth Journey* intervention*. Childbirth Journey* is a digital support tool for parents developed in Sweden by healthcare professionals in antenatal, labour, postnatal and child health care; by a researcher specialising in serious games; and by a game development company [for more details, please see (15)]. *Childbirth Journey* is designed to help expectant parents acquire more knowledge about how to prepare for childbirth and parenthood. *Childbirth Journey* is available as both a mobile game and a stand-alone PC application. The mobile game version was explored in the present study.

*Childbirth Journey* consists of two distinct parts: (1) a story-driven game and (2) a *Knowledge Portal,* described below.

The story-driven game provides a narrative experience, which is intended to stimulate the user's interest continuing with the game. The *Childbirth Journey* narrative helps players experience the process of birth preparation, the onset of labour contractions and actual birth at a maternity ward in a Swedish hospital environment. The story is divided into three parts: *Home, preparation*; *Home, labour starts*; and *Labour, at hospital.* These parts were explored in the current study. Further scenes—titled *Midwife antenatal clinic*, *Postnatal ward at hospital, Home after childbirth* and *Midwife postnatal clinic*—are planned for development in a future version of *Childbirth Journey.* The different scenarios (the expectant parents' preparation for childbirth, the onset of labour, labour, pain relief, breastfeeding, formula, parental leave and parenthood) and associated information provided in *Childbirth Journey* are based on evidence as well as professional knowledge and actual routines applied at antenatal units and at the labour ward at the targeted hospital. In *Childbirth Journey,* the player can influence a scenario by choosing among the various options presented, thereby allowing events to unfold differently depending on which options are chosen. The information in the game is presented in text format, and digital speech synthesis is optional. As a complement to the game, several films lasting from between 5 and 15 min were developed to depict various conversations between healthcare professionals and parents as well as to illustrate the various examinations and procedures an expectant parent can expect to undergo during labour. The healthcare professionals who took part in the films were all working in the labour ward at the hospital where the films were recorded. These films can also be accessed directly through the *Knowledge Portal,* as described below. In addition, in the story-driven game, different weblinks to external websites appear depending on the choices made by the player to provide the user with access to additional information [for more details, please see (13)].

The *Knowledge Portal* serve as a library of links to external websites that might be of interest to expectant parents: a Swedish national website providing health information (www.1177.se), a resource on Swedish social insurance issues (www.forsakringskassan.se), links to podcasts and literature suggestions in book or article format. The *Knowledge Portal* provides information regarding pregnancy and labour, i.e., the female body during and after pregnancy, pain relief, breathing techniques, breastfeeding and formula, baby care, the parental role, parental leave, and the parental couple relationship. In addition, the films included in the story-driven game are also traceable through the *Knowledge Portal* [for more information on the *Knowledge Portal*, please see (13)].

In the current study, healthcare professionals were given access to *Childbirth Journey* via a download. They were instructed to use *Childbirth Journey* according to their own needs and interests but also to test all three scenarios in the game and to visit the *Knowledge Portal*.

### Settings and participants

The setting for the present study was representative of the Swedish population, including both rural and suburban residents. Approximately 280,000 people reside in this setting, which has 15 antenatal units, one hospital with a labour and postnatal ward, and 28 child healthcare units. Participants were recruited using convenience sampling: Social media channels were used to advertise the opportunity to participate in the intervention to healthcare professionals, heads of antenatal units and labour and postnatal wards, and child healthcare unit staff within the research setting. Specifically, the advertisement asked healthcare professionals (midwives and specialist nurses working at child healthcare units) about their interest in participating in the study. In total, 11 healthcare professionals (seven midwives and four specialist nurses) agreed to participate. They were provided with further information about the study from the first author and subsequently received the intervention. The participants were requested to use all the functions in the game, as different choices and test several scenarios as they choose, as well as explore the Knowledge Portal. The participants were between 37 and 66 (mean 46) years old and had between 2 and 41 (mean 15) years of work experience as midwives or specialist nurses in child health service. Three of the participants worked in antenatal units within the research setting, three worked in the labour ward, one worked in the postnatal ward, and four worked in child healthcare units. All of the participants used the mobile application in the Swedish language.

### Data collection

Data were collected through semi-structured interviews based on an interview guide that included both open-ended and follow-up questions, such as *Could you tell me about your perceptions of the Childbirth Journey? Which parts of the Childbirth Journey did you appreciate more than others? Were any parts of the Childbirth Journey difficult to understand?* and *Will you recommend the Childbirth Journey to expectant parents you care about?* Because of restrictions imposed on in-person social interactions due to the current COVID-19 pandemic, all of the interviews were held virtually, with a video link so that the participants and researchers could see each other during the interviews. Two of the researchers (CB & ML) held three focus-group interviews with three or four participants and one individual interview. At the time of the interviews, the participants had already had access to the *Childbirth Journey* intervention for approximately two weeks. The interviews were audio-recorded and lasted between 23 and 75 min (mean 49 min).

### Data analysis

The seven steps described by Sjöström and Dahlberg (17) were used for data analysis. To gain a more thorough understanding of the phenomenon healthcare professionals' perceptions of Childbirth Journey as a digital tool in the data material, in the first step (familiarisation), the transcripts (46 A4-pages, 1.0-spaced type) were read repeatedly and discussed by the authors. Afterwards, in the second step (compilation), the most significant parts of the transcripts—i.e., those parts that addressed the aim of the study—were identified. Then, in the third step (condensation), comparisons were made in order to identify similarities and differences related to participants experiences of the phenomenon, which were then grouped and related to each other in the fourth step (grouping). To identify distinct boundaries between groups, further comparisons between similarities and differences were made, representing a fifth step (comparison). In this step, all authors mutually reflected on these patterns and distinctions, resulting in the emergence of four descriptive categories, which were identified in the sixth step (naming). Finally, in the seventh step (contrastive comparison), comparisons were made to identify the logical relationship between the descriptive categories. The authors had equivalent and complementary experience in qualitative methods and phenomenographic analysis, and, as such, had a mutual understanding of the analysis of the interviews. During the analysis process recurring discussion secessions were organize in order to achieve consensus among the authors.

### Ethical considerations

The Regional Ethical Review Board in Gothenburg, Sweden, approved this study (Dnr: 2020-01689). The intervention was considered to be low-risk. Nevertheless, some of the participants may have had negative experiences related to, for instance, the use of *Childbirth Journey* or more general technological problems. This may have led these participants to experience frustration or disappointment, potentially leading to dropouts from the study.

Before potential participants consented to participate, they received study information in both written and verbal form. They were also given the freedom to choose the time at which they would be interviewed. The audio-recorded interviews were securely stored, with access limited exclusively to the researchers. The participants' identities were kept confidential, and, accordingly, the interview excerpts presented in the Results section do not include names or other personally identifiable information.

## Results

### Descriptive categories

The perceptions of healthcare professionals, midwives and child healthcare nurses about *Childbirth Journey* are presented in four descriptive categories: *Extended professional support, Trustworthy contents, Diversity or individuality,* and *Both appealing and in need of development*. Each descriptive category is emphasised in the text by using direct quotes (translated from Swedish to English) from the healthcare professionals. In the following, the term “*parent”* refers to either an expectant parent or a parent with a newborn child.

#### Extended professional support

The participants described *Childbirth Journey* as an extended opportunity for them to provide parents with information when they needed it, which, according to the participants, was a good way to use a digital solution to support parents. The information in *Childbirth Journey* was perceived by the participants to be congruent with the information they give to parents in the course of their everyday clinical work. To them, *Childbirth Journey* represents an extended opportunity for parents to obtain information that accords with the information they themselves provide. The participants reflected on the potential to rely on *Childbirth Journey* as a digital support tool for parents to formulate—in advance—important questions to ask healthcare professionals during care meetings. Some of the midwives in antenatal care service described how, during care meetings, expectant parents sometimes did not know what to ask them about:

… if they [parents] look in Childbirth Journey and at the different alternatives and descriptions that exist, they [parents] get answers to their questions, and it may not always be so strange or complicated questions either, but how should I say now, sometimes they [parents] do not really know what they should ask about (Int 11).

The participants mentioned that *Childbirth Journey* allows parents to repeatedly play the game and rely on the *Knowledge Portal* to obtain relevant information. Moreover, *Childbirth Journey* gives partners the opportunity to obtain vital information even when they are unable to participate in meetings at the antenatal or labour ward or other child healthcare service (primarily as a consequence of the COVID-19 pandemic). The participants appreciated how parents were able to obtain relevant advice about how to manage various situations—for example, at home during the latent phase of labour—when using *Childbirth Journey*. As one midwife described:

I'm thinking of the preparation at home. Where you also get to see a little closer that you do not have to go in [to the hospital] immediately. That you [parents] can actually do a lot at home and that you [parents] can still feel safe, and that the partner and the woman can work very, very much together for it to work well before they make the decision to go [into the hospital] (Int 10).

#### Trustworthy contents

The information in *Childbirth Journey* was described by the participants as objective and trustworthy—and in accordance with current national and local guidelines for antenatal, labour, and postnatal care. In the game, the provided information was perceived as reasonably comprehensive and clear. The participants also noted the value of the “logical thread” that was illustrated in both the game and the *Knowledge Portal:* pedagogically described natural processes occurring during pregnancy, labour and breastfeeding. The participants believed that such pedagogy would help parents better understand such natural processes, such as the ways in which a woman's body physically changes during pregnancy, labour and breastfeeding. The *Knowledge Portal* was described by the participants as a highly valuable base of knowledge. Further, they appreciated the varied ways in which parents could collect information through the *Knowledge Portal*—e.g., receiving tips about helpful literature and podcasts as well as links to the websites of other authorities and organisations:

… Some may want to read up on information. Another may want to listen to podcasts. It's probably a little different how it is I think. What's interesting (Int 11).

The films included in *Childbirth Journey* were perceived as being of good quality and consisting of trustworthy information because such information was objective and delivered by healthcare professionals working in the labour ward of a real clinical hospital. The healthcare professionals (i.e., midwives and obstetricians) in the films were perceived as being calm and as possessing expert and reliable knowledge concerning pregnancy and labour:

… films that it is very, gives a very safe impression. I think that by seeing it and knowing that you are going to give birth at the ward, you can probably just calm down by understanding that it is that atmosphere (Int 10).

In the game, the participants perceived that the trustworthiness of the content was strengthened by the graphical environment, which mirrored an actual labour room in the labour unit of the local hospital. This was understood to be particularly positively as the parents would easily recognise the labour unit when they first arrived at the labour ward after playing the game. That said, some limitations of the game were mentioned as well. For example, the midwives working in labour care believed that the trustworthiness of *Childbirth Journey* decreased when the game illustrated only two professionals (i.e., two avatars) in the labour room at the moment of birth—namely, two midwives—when, in reality, three professionals are present: two midwives and an assistant nurse. These participants expressed concern that the omission of assistant nurses from the game would frustrate these nurses and present an inaccurate depiction of the labour and birth process.

#### Diversity or individuality

The participants appreciated that, in the game, the avatars were gender-neutral and multicultural in appearance. For example, the professionals had names like Kim, Billy and Charlie, and the pregnant woman had a neutral skin tone. The avatars for both the healthcare professionals (midwives and assistant nurses) and partners had an androgyne appearance. The participants recommended that, in a future version of *Childbirth Journey*, it would be helpful if the player could select between different avatars to use in the game. They asserted that partners should feel welcome to use the game, regardless of gender, and, for this reason, different avatars were needed:

I understood … because the partner's name is Anna, the one who calls [into the hospital] and so on too, but I think it would have been good to be able to choose whether the partner should be a man or a woman, or, it does not have to be especially so, but to be able to choose between a few different people, to include everyone (int 4).

The participants perceived that the identity of the family is a subjective experience, one that should be affirmed in the game. To do so, future versions of *Childbirth Journey* could include extended opportunities to choose with which avatar to play, including those with both masculine and feminine characteristics:

That you can choose, because there are so many different constellations in the families and because everyone should feel included, it would have been good if you could, that there is a choice (Int 4).

#### Both appealing and in need of development

The participants described aspects of *Childbirth Journey* that they found to be appealing, as well as features they felt were in need of further development. When the game design facilitated a better understanding of how to manage specific parts of the game, they found it to be appealing. For example, the “i” button (i.e., “i” is a shortcut to the *Knowledge Portal*) available in the game made it easier for the participants to quickly switch between playing the game and accessing information in the *Knowledge Portal*. They also found the following features to be appealing: the freedom to make their own choices in the game, controlling and moving the avatars, and clicking on items in the game environment to obtain information.

As noted by the participants, several aspects of *Childbirth Journey* were in need of further development. For example, the participants found some parts of the game to be difficult to understand, particularly what actions to take to progress in the game. Some participants had to resort to asking their children (about 12 years old) for help in playing the game. This is likely because children have more experience playing digital games. As such, these participants questioned whether players would need to have gaming experience to be able to navigate *Childbirth Journey*. Concerning the audio dimensions of the game, the participants found the recorded human voice to be calming, whereas the digital speech syntheses, the digital voice, sometimes incorrectly pronounced words and sounded impersonal. This in turn made it difficult for some participants to understand what was being communicated, although others had no such issues. The participants also noted that they were unable to regulate game audio without restarting the application. Occasionally, game sounds would change spontaneously, from high to low, and, on other occasions, sounds were missing altogether, which caused frustration. Also, the participants expressed the desire to have the opportunity to listen to the information provided in the *Knowledge Portal* (a speech synthesis)*—*in the current version, this information is only available in a read-only format:/

Yes, a robotic voice; it sounded like there were such misemphasis and, yes, it almost felt a bit parodic at times. It almost did not feel—yes it did not feel serious in any way … yes it would probably have been better if it had been real people, so to speak, who answered it (Int 9).

The participants felt that the lighting and colours in the game should be changed to make them more representative of the real environmental conditions of the local hospital labour ward. Similarly, the home environment as depicted in the game was experienced as looking more like a hotel room than an actual home.

The films were perceived to be appealing, with a trustworthy and serious impression, but the game graphics and functions were regarded as being in need of further development to correspond better with the level of quality that healthcare professionals seek to maintain when meeting with parents. The 3D graphics were described as awkward and clumsy, and it was recommended that the *Knowledge Portal* would benefit from a more appealing design and more secure functions, as some of the weblinks did not work.

In the game, the avatars representing healthcare professionals would, on occasion, be placed in an incorrect position in relation to the avatars representing the parents. This, the participants felt, could raise concerns among some parents who played the game as it would give them an inaccurate impression of how healthcare professionals position themselves in relation to parents during care meetings. Thus, the participants recommended that the avatars be positioned in such a way that midwives were clearly shown to provide manual perineal protection during the moment of birth and how the newborn children are actually handed over to the mothers.

Sometimes, the buttons intended to allow players to make choices in the game were positioned outside of the screen, consequently making them unable to use, which caused frustration among the participants and adversely impacted their assessment of the trustworthiness of *Childbirth Journey* overall. In addition, when using a mobile device to play the game, the game text was too small to read. The participants therefore requested additional functions that would allow them to go back and forth in the game and, also, the option to change their choices in the game without being forced to restart it from the beginning. They additionally asked for the ability to change player perspectives, from pregnant to partner, or vice versa:

… I switched between different perspectives in the labour moment. The midwife and the partner talked and then the midwife answered back as if she was answering the pregnant woman. It was a bit like using the same text as you did when playing the pregnant. So that made me a little confused (Int 4).

To obtain extended information, the participants requested increased opportunities for the player to interact more with various items in the in-game spawning environment. They suggested that future game developments permit players to explore the medicine cart or the table on which birthing instruments were placed, for example. Furthermore, they asked for more relevant and comprehensive information applicable both during pregnancy and in the first moments spent with a newborn child. In the current version of *Childbirth Journey,* the main focus is on labour. Consequently, some participants requested more information about antenatal care, vitamin K for newborns, child–parent attachment and breastfeeding, as well as more information about parental couple relationships and family centres. The participants reflected on the possibility of using humour to convey information to parents in a more pleasurable fashion:

… the information was great. Good lyrics // So I certainly think it fulfils a function, but if you could get a little fun angle on the whole thing, then I think it would be even better (Int 7).

Participants working within labour care were especially frustrated by the graphical limitations of the game. Due to these limitations, they could not recommend *Childbirth Journey*—in its current format—to parents. This is because these participants were worried that parents would have inaccurate impressions of labour units and care due to graphical inconsistencies and limitation and would, consequently, make them more anxious. The participants thus suggested that more films be included—for example, films that depicted the moment of birth with the midwife accurately shown providing manual perineal protection, in addition to added illustrations of various birth positions and how newborns are actually handed over to their parents. Furthermore, these participants requested that an assortment of pictures be included for the purpose of better visualising specific environments (such as an examination room), items (such as a CTG device) and situations (such as a labour assisted with a vacuum extraction).

Overall, the participants agreed that *Childbirth Journey*, given some improvements, could be used as an effective digital support tool for parents, one which would allow healthcare professionals to offer such digital solutions as a complement to standard, face-to-face meetings with caregivers. According to the participants, *Childbirth Journey* could be used to expand and refine parents' knowledge as the game includes relevant, evidence-based knowledge in a mobile device format. The participants appreciated the fact that *Childbirth Journey* was being developed as a digital support solution that would be free of charge for parents and easily accessible for both parents and healthcare professionals. Although the participants considered *Childbirth Journey* to be very promising for Swedish- and English-speaking parents, those who speak other languages, such as Somali and Arabic, would need to have access as well, thereby necessitating language-based adjustments to future versions of the game for the sake of inclusivity and representativeness.

### The outcome space

The outcome space summarises that *Childbirth Journey*, with some improvements, can be used as a digital support tool for parents, allowing healthcare professionals to offer such digital solutions as a complement to standard, face-to-face meetings with caregivers. The results of this study reflect a hierarchical relationship between the descriptive categories, as described in the following. Concerning the first descriptive category, *Extended professional support,* the participants felt that *Childbirth Journey* could be offered as a complement to standard care, permitting professionals utilizing a digitally based support solution for support to parents. Towards this end, *Childbirth Journey* should include *Trustworthy contents* (second descriptive category) and allow for both *Diversity and individuality* (third descriptive category) in terms of content. Perhaps most importantly, for *Childbirth Journey* to function as a digital solution for extended professional support (first descriptive category) to parents in the future, further developments—such as those described above—are needed. That said, the current version *does* possess multiple appealing features—i.e., the fourth descriptive category, *Both appealing and in need of development*.

## Discussion

The WHO has underscored the importance of monitoring and reporting on the development of digital innovations in public health (18), and serous game is rapidly growing area within healthcare (12). Hence, this study on the perceptions of healthcare professionals about digital support for expectant and new parents contributes valuable knowledge to this research domain. The study encompassed cross-professional perceptions since both midwives charged with various aspects of care for expectant and new parents and child healthcare nurses were included. This decision could be considered a strength of this study since various healthcare professionals, all caring for new families, were included in the exploration of the intervention. Although, as the results show, the participants perception of the *Childbirth Journey* were similar in one aspect they had different opinion. This applies to recommendation of *Childbirth Journey* in this present form.

The current study was part of a larger research project (15), which covers different steps of development for digital supports for parents and associated explorations. The intervention in this study, *Childbirth Journey,* is planned for development in two steps. In the first step, parents (13) and healthcare professionals (current study) use the intervention and exploration on their perceptions of it is included. In the second step, further developments of the *Childbirth Journey* intervention will be performed based on the research findings from the first step. Hence, both target groups of users (i.e., parents and healthcare professionals) are being included in the development, and exploration, of the intervention*.* Previously, both the need for developing digital supports for parents (7) and the inclusion of healthcare professionals in such developments (14) have been stressed. Stevenson et al. (14) noted that involving midwives in the development of digital sources can provide the opportunity for content to be designed to meet specific user needs in practice.

The results from the current study highlight several strengths—but also some limitations—of the intervention, *Childbirth Journey.* The participants perceived, for example, that the information included in *Childbirth Journey* represents a trustworthy knowledge base from which parents can deepen and refine their knowledge of natural processes during pregnancy, labour and breastfeeding. The information included is also, according to the participants, in line with current guidelines, and, altogether, could contribute to enhancing parents' awareness of how to prepare for both birth and care meetings. The importance of professional support in facilitating the improvement of parents' knowledge and preparation has been asserted in several research studies (19), and, according to our findings, healthcare professionals consider *Childbirth Journey* to be a promising future complement to regular professional support in antenatal, labour and postnatal care. This finding is in line with research on parents’ perceptions of *Childbirth Journey* which conclude it is a valuable digital complement to in-personal professional support (13), and meets the Swedish National Board of Health and Welfare's call for antenatal care to offer alternative forms of support to parents, such as digital educational materials (7).

Even though the current findings revealed several strengths and benefits of *Childbirth Journey* as a future digital parental support tool, several concerns were raised that should be considered in future developments. Overall, the participants found the information included in the intervention to be satisfactory and trustworthy. Further, the concept of creating a serious game that narratively illustrates a labour environment in ways faithful to actual conditions at local hospitals was perceived positively since this approach could broaden parents' understanding of what to expect when arriving at the hospital and permit them to plan and prepare accordingly. However, the game graphics would benefit from further developments, including updating them with more modern, professional and accurate nuances. Additional functions that would allow players to move back and forth in the game more easily were also recommended by participants, as were more opportunities to choose the characteristics of the avatars in the game. The participants had a positive view of the gender-neutral and multicultural appearance of the present avatars but felt that such features could be expanded in the future, with more options. The positioning of the avatars also requires further developments, especially the positioning of midwives working within labour care (e.g., the midwife avatar did not provide manual perineal support during the moment of birth), which the participants who worked as midwives felt was not inaccurate and therefore untrustworthy. The considerable number of identified limitations attributable to graphics, sounds and other technical functions led some participants (mostly midwives working within labour care) to decline to recommend *Childbirth Journey* to parents in its current format. Their reasoning for this decision was that they were worried that parents who used *Childbirth Journey* would get an inaccurate impression of actual labour unit practices, which might in turn make these parents more anxious (13, 20). This revelation highlights the importance of having a development team with broad experience, one that includes both healthcare professionals with clinical knowledge and technical staff that possess expertise in building applications and games—importantly, this team should be collaborative and well-integrated.

Stevenson et al. (14) asserted that including midwives in the development of digital support tools would increase the likelihood that they would adopt and implement such applications in their future work. This is valuable to consider when developing digital support interventions and other tools with sustainability in mind. This research project (15) is being conducted in two steps, as previously described, with further developments of *Childbirth Journey* being based on the findings of the current study, among other sources (13). Another reason to include healthcare professionals in the development and exploration of digital supports is educational in nature. Jevitt, Houston, Anderson, Ku Carbonell and Abdul (21) claimed that there was an urgent need for digital learning among midwives. This is because when midwives do not have sufficient time to learn new digital skills, a digital divide can be created between them and the women, families and communities they support. Such a divide may risk digital burnout among midwives. To minimise such a risk, investments in digital midwifery and appropriate training should be provided, as doing so would ensure that they have a better understanding of how to provide the best support for clients. This may also be relevant to other professions, such as specialist nurses in child healthcare services. In another study, Vivilaki and Asimaki described how midwives should become the strategic guardians for women in the ongoing digital transformation in our society (22) so that they can succeed in this digital transformation (9). This observation was also made in another study that problematised whether parents were truly in need of a digital cicerone in health care to guide them through the sensitive phase of childbearing and early parenthood (20). In our findings, the participants described experiencing frustration with technical errors or insufficient guidance on how to proceed in the game. One participant mentioned how she had to consult her 12-year-old child on how to manage the game, which the child was quite capable of doing. This highlights how valuable digital learning and skills are among healthcare professionals (21). It also emphasises the value of having the right opportunities to build a digital support tool that is user friendly, a prerequisite for which may be the financial aspect, which was strictly limited in the current study. The risk of generating frustration among healthcare professionals when introducing them to new digital support tools is that they might be hesitant to recommend these tools to their clients. Consequently, investing financial resources in building such tools would not be sustainable. However, the results of the current study demonstrated the potential for *Childbirth Journey* as a *future* digital support tool, one that could expand and refine parents' knowledge. Moreover, the participants noted the importance of being able to confidently recommend *Childbirth Journey* as a digital support tool for parents so long as it includes professional, evidenced-based knowledge in line with current guidelines and organised in a single, easily accessible digital arena.

## Conclusion

Developing a healthcare digital tool for parental support in the form of a serious game is a complex process, one that requires assessing and incorporating the experiences of both parents and professionals. The results of the present study demonstrated that digital parental support can provide reliable information and may represent a valid form of extended professional support. Nevertheless, the evaluation of such digital parental support from the perspectives of professionals contributes valuable feedback that must be considered as well, such as the possibility that these digital tools may worsen anxiety in parents. According to the basic ethical principle in medicine, *primum non nocere,* pilot projects such as ours are necessary in order to evaluate interventions in the pre-final phase—particularly their potentially adverse consequences. This, then, is the main contribution of our study: evaluating the strengths and weaknesses of a digital support tool for expectant parents and assessing its present and future utility outside of the framework of the present study.

## Data Availability

The original contributions presented in the study are included in the article/Supplementary Materials, further inquiries can be directed to the corresponding author.
